# Effect of masticatory training using confectioneries on oral function in elderly patients – A randomized controlled trial

**DOI:** 10.1016/j.jds.2022.04.030

**Published:** 2022-05-20

**Authors:** Hitomi Nishizaki, Takatoshi Iida, Yohei Tanaka, Yoshinari Morimoto, Megumi Hayashi, Lou Mikuzuki, Yuki Yao, Yuichi Tatsuno

**Affiliations:** a Department of Geriatric Dentistry, Kanagawa Dental University, Yokosuka, Kanagawa, Japan; b Department of Speech-Language-Hearing Therapy, School of Rehabilitation Sciences, Health Sciences University of Hokkaido, Ishikari, Hokkaido, Japan

**Keywords:** Confectionery, Elderly patient, Masticatory ability, Masticatory training, Occlusal force

## Abstract

**Background/purpose:**

The number of patients with oral hypofunction is increasing with the aging of the population, and such hypofunction increases their risk for dysphagia and malnutrition. The purpose of this study was to measure the hardness of commercially available confectioneries, select a confectionery with a hardness suitable for masticatory training for elderly patients, and evaluate the effects of 1-week masticatory training on oral function (occlusal force, masticatory ability, and tongue pressure).

**Materials and methods:**

The average hardness values of 25 confectioneries were determined. Among them, one of the softest confectioneries that the patients felt as “chewable but difficult to chew” was selected as the training confectionery for each patient. The patients in the training group continued training, which involved eating of approximately 5 g of one selected confectionery daily for 7 days. The patients in the control group did not undergo any training. Oral function (occlusal force, masticatory ability, and tongue pressure) on the first day and after 7 days was evaluated and compared between the groups.

**Results:**

The occlusal force of the patients in the training group increased significantly. However, their masticatory ability and tongue pressure did not change significantly.

**Conclusion:**

Patients aged 65 years and older underwent masticatory training, which involved eating of a confectionery with its hardness adjusted individually for a week. A significant increase in the occlusal force was observed, suggesting that masticatory training using confectioneries with a hardness suitable for each patient is effective.

## Introduction

In Japan, the number of patients with oral hypofunction is increasing with the aging of the population, and such hypofunction increases their risk for dysphagia and malnutrition.^1^ When the masticatory ability declines, foods that are difficult to chew are avoided, while foods that are soft and easy to chew are selected.^2^ Water is often added to adjust and consequently soften the form of food, which increases the volume of food. As a result, the energy from food becomes less than half per volume compared with that prior to morphological adjustment, and the adjusted diet may not be able to supplement the necessary nutrition, worsening the nutritional status of elderly patients.^2^^,^^3^ Therefore, training to improve their masticatory ability is important.^4^

In previous studies, various masticatory training programs with hard foods have improved the masticatory ability in young or middle aged individuals; however, hard foods that can be chewed daily with the maximum occlusal force cannot be tolerated among elderly patients with decreased masticatory ability.^5^, ^6^, ^7^, ^8^, ^9^, ^10^, ^11^, ^12^ Since there are large individual differences in mastication, adjusting the properties of the food used for masticatory training is necessary to achieve a load suitable for each elderly patient.

The purpose of this study was to measure the hardness of commercially available confectioneries, select a confectionery with a hardness suitable for masticatory training for elderly patients, and evaluate the effects of 1-week masticatory training on oral function (occlusal force, masticatory ability, and tongue pressure).

## Materials and methods

### Study population

This randomized controlled trial was conducted in accordance with the Declaration of Helsinki and was approved by our institutional research board and ethics committee (approval number: 614). The study process and procedures were explained to all participants, and written informed consent was obtained. This study was registered in the UMIN Clinical Trials Registry (UMIN000044947, registered on July 24, 2021).

The participants were outpatients and home-visit patients who visited the dental clinic of our hospital from July 2021 to March 2022. Patients aged 65 years or older with a Dysphagia Severity Scale (DSS) score of 3 (water aspiration) or higher were included in the study.^2^^,^^13^ The participants were randomly assigned to two groups using a computer: patients who underwent masticatory training using a confectionery with a hardness adjusted to the individual masticatory function (training group) and patients who did not undergo masticatory training (control group). Patients with food allergies and an insufficient understanding of the instructions owing to the presence of cognitive impairment were excluded.

### Selection of training confectionery

Twenty-five commercially available confectioneries (rice crackers and biscuits) were provided. The hardness of these confectioneries was measured using a creep meter (RE2-33005C/Yamaden, Co., Ltd., Tokyo, Japan) based on the universal design food (UDF) product test method.^14^ UDFs are defined by the Japan Carefood Conference (JCC) as processed foods and foods used for adjusting the shape, physical properties, and container to make them easier to eat according to the user's masticatory and swallowing abilities.^14^

The method of measuring the physical properties (hardness and adhesiveness) of the UDFs was determined in accordance with the standard regulations of foods for individuals with difficulty in swallowing indicated by the JCC.^14^ This method allowed the measurement of the relationship between stress and strain by lowering the plunger horizontally from the top surface to the sample at a constant speed and pressing it twice.^14^, ^15^, ^16^

In this study, we measured the hardness (N/m^2^) using the maximum force required for the first compression. The measurement conditions were as follows: load cell of 20 kg, compression rate of 10 mm/s, and plunger diameter of 3 mm. Because the test food could not be transferred to the measurement container, the clearance was set to 30% of the sample thickness, and the measurement temperature was set to 20 ± 2 °C. The measurements were performed five times per sample, and the average values of the three values, excluding the minimum and maximum values, were calculated.^14^, ^15^, ^16^ The average values of the hardness of the 25 types of confectioneries were arranged in order of softness, and the confectioneries with the minimum value of hardness, first quartile value, median value, third quartile value, and maximum value were selected as the training confectioneries. The patients in the training group were fed each confectionery in a soft to hard order before the start of training. Among the three choices of “chewable without difficulty,” “chewable but difficult to chew,” and “not chewable,” one of the softest confectioneries that the patients felt as “chewable but difficult to chew” was selected as the training confectionery for each patient.

### Intervention methods

The patients in the training group continued training, which involved eating of approximately 5 g of one selected confectionery daily for 7 days. There were no restrictions on the eating time or meals other than those used during training. The patients in the control group did not eat any confectionery and were instructed to live and eat as usual for 7 days. The first measurement (standard value) was performed on the first day and the second measurement after 7 days. Based on the results of the preliminary survey, the training duration was limited for a week because many participants claimed fatigue and refused to eat a confectionery with a hardness that was adjusted individually for more than a week.

### Survey items

#### Patient background

Age, sex, and complications were investigated via a medical record review. At the beginning of the study, the Eichner classification, number of occlusal support areas with prosthetics, and DSS score were assessed.

#### Occlusal force

The participants were instructed to bite on the pressure-sensitive sheet of Dental Prescale II® (GC Co., Ltd., Tokyo, Japan) in centric occlusion for approximately 3 s, and the occlusal force was measured. The value measured in the first measurement was used; the value in the second measurement was used only when the first measurement failed. Less than 500 N of occlusal force was defined as a decreased occlusal force.^17^ The denture users underwent the measurement with their dentures mounted.

#### Masticatory ability

The masticatory ability was evaluated using 2 g of glucose-containing gummy jelly (Glucorum®, GC Co., Ltd.,; gummy jelly). The value measured in the first measurement was used; the value in the second measurement was used only when the first measurement failed. A glucose concentration of <100 mg/dL was considered to indicate masticatory hypofunction.^18^

#### Tongue pressure

The tongue pressure was measured using a tongue pressure-measuring device (JMS tongue pressure-measuring device TPM-02®, JMS Co., Ltd., Tokyo, Japan). Three measurements were performed, and the average value was used as the measured value. Less than 30 kPa of tongue pressure was defined as a low tongue pressure.^18^

### Statistical analysis

The measured values were expressed as medians (quartiles). The Mann–Whitney U test was used to compare patient age and sex. Fisher's exact test was used to compare the Eichner classification, number of occlusal support areas, and DSS score. The Smirnov–Grubbs test was used to test for outliers. The Wilcoxon signed-rank test was used to assess the differences in the occlusal force, masticatory ability, and tongue pressure before and after training. The significance level was set at *P* < 0.05.

The method for determining the required number of cases was calculated on the basis of a previous report.^6^ When calculated with an α error of 5% and β error of 20%, the required number of cases was 21 in each group (42 in total). Since the patients were enrolled until the number of patients in both groups reached 21, there were 23 patients in the training group.

## Results

### Patient background

**Figure 1:**
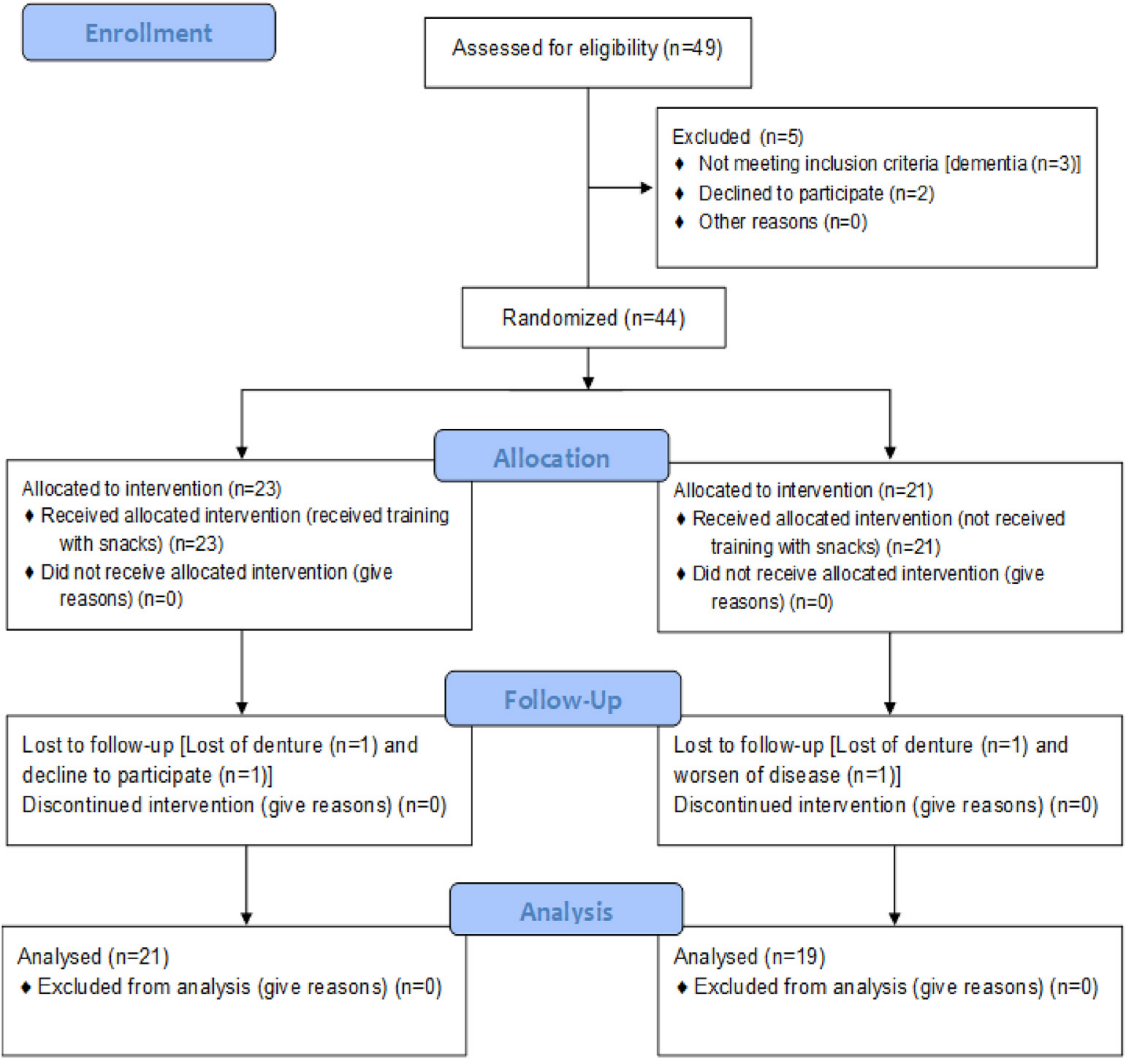
Consolidated Standards of Reporting Trials flow chart showing the patient selection process. Forty patients were included in this study.

Forty-four patients were enrolled in the study, of whom total of four withdrawn (Fig. 1). Thus, 40 participants were finally included in the analysis. There were no significant differences in age, sex, Eichner classification, or number of occlusal support areas between the two groups (Table 1, Table 2). Regarding swallowing function, there was no significant difference in the distribution of the DSS score between them (Table 2).

**Table 1:** Characteristics of the participants.

Item	Training group (n = 21)	Control group (n = 19)
Patient background		
Age	77.0 (73.0–80.0)	80.0 (76.0–85.5)
Sex (M/F)	10/11	11/8
Outpatients	12	13
Home-visit patients	9	6
Disease (duplicate)		
Stroke	7	7
Hypertension	8	7
Arrhythmia	2	3
Ischemic heart disease	0	1
Aortic stenosis	1	0
Hypertrophic cardiomyopathy	0	1
Rheumatoid arthritis	1	0
Parkinson's disease	0	2
Osteoporosis	3	3
Diabetes mellitus (type 2)	1	3
Others	3	7
None	2	1

The measured values are expressed as medians (quartiles).

**Table 2:** Conditions of the participants.

Item	Training group (n = 21)	Control group (n = 19)	*P*-value
Eichner classification^a^			0.199
A	7	3	
B	3	12	
C	11	4	
Number of occlusal support areas^b^			0.223
4	19	16	
3	1	3	
2	1	0	
Dysphagia Severity Scale score^c^			0.277
3	2	3	
4	2	4	
5	1	1	
6	1	4	
7	15	7	

^a–c^Fisher's exact test.

### Training confectionery

**Figure 2:**
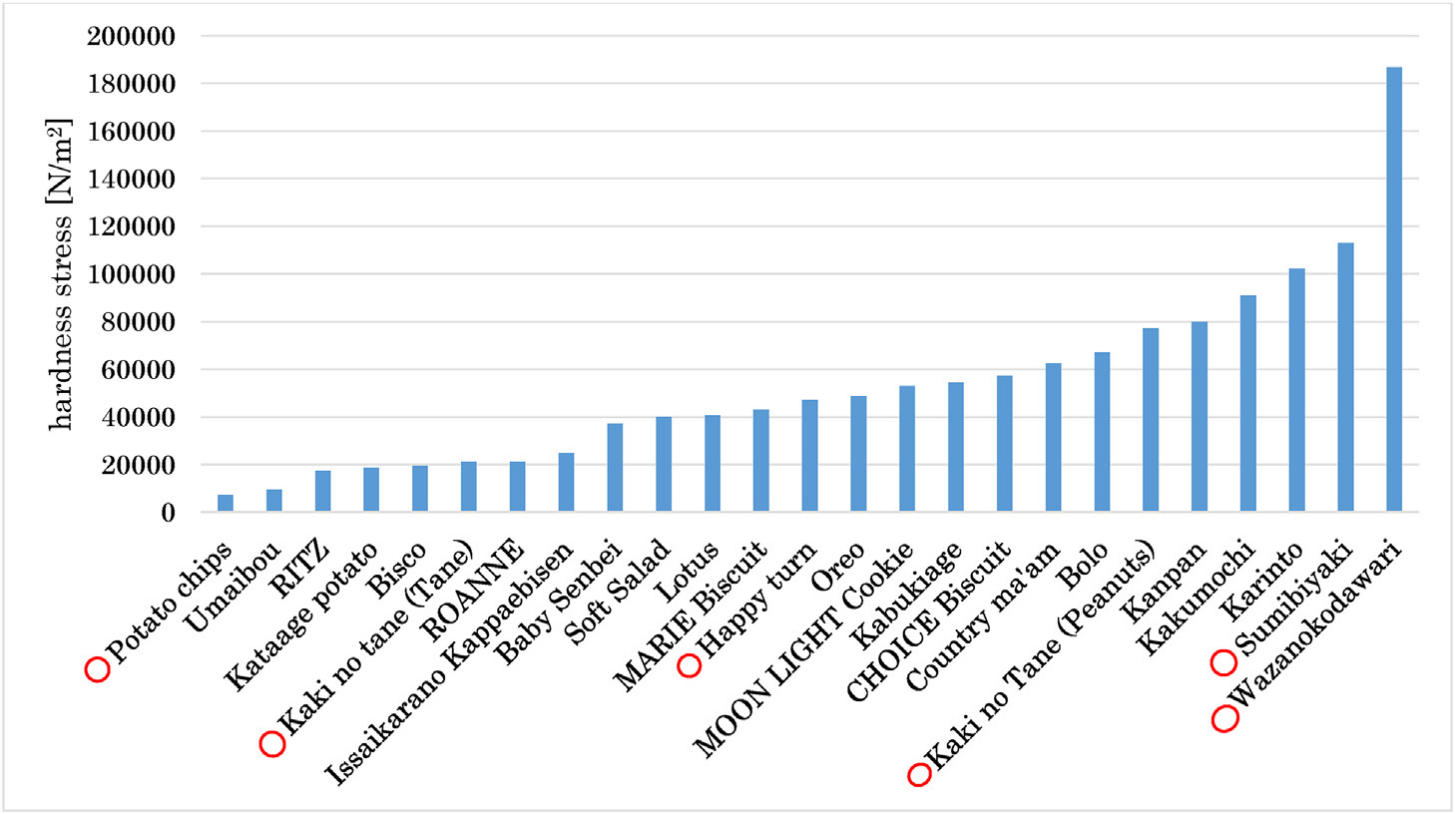
Distribution of confectionery hardness. The average value of the hardness of 25 types of confectioneries was calculated, and a graph arranged in the order of softness was suggested. Regarding hardness, the minimum value (Potato chips®, 7388 N/m^2^), first quartile value [Kaki no tane (Tane)®, 21,185 N/m^2^], median value (Happy turn®, 47,240 N/m^2^), third quartile value [Kaki no Tane (Peanuts)®, 77,206 N/m^2^], and maximum value (Wazanokodawari®, 186,773 N/m^2^) were selected. The maximum hardness of the confectionery (Wazanokodawari®) was an outlier (*P* = 0.0027), followed by the second most difficult to chew confectionery (Sumibiyaki®, 112,896 N/m^2^) (*P* = 0.4688). Red circles indicate the adopted confectioneries.

The average hardness value of the 25 types of confectioneries was calculated, and a graph arranged in the order of softness was suggested (Fig. 2). The minimum value (Potato chips®, KOIKE-YA Inc., Tokyo, Japan; 7388 N/m^2^), first quartile value [Kaki no tane (Tane)®, Kameda Seika, Co., Ltd., Niigata, Japan; 21,185 N/m^2^], median value (Happy turn®, Kameda Seika, Co., Ltd., Niigata, Japan; 47,240 N/m^2^), third quartile value [Kaki no Tane (Peanuts)®, Kameda Seika, Co., Ltd., Niigata, Japan; 77,206 N/m^2^], and maximum value (Wazanokodawari®, Kameda Seika, Co., Ltd., Niigata, Japan; 186,773 N/m^2^) were selected. In the Smirnov–Grubbs test, the maximum hardness of the confectionery (Wazanokodawari®) was considered an outlier (*P* = 0.0027). The second most difficult to chew confectionery (Sumibiyaki®, Ando Seika, Co., Ltd., Tokyo, Japan; 112,896 N/m^2^) (*P* = 0.4688; this was not an outlier) was also added, and six types of confectioneries were prepared. The patients in the training group were fed six types of confectioneries in a soft order before the start of training. Among the choices of “chewable without difficulty,” “chewable but difficult to chew,” and “not chewable,” one of the softest confectioneries that the patients felt as “chewable but difficult to chew” was selected as the training confectionery.

### Oral function

**Figure 3:**
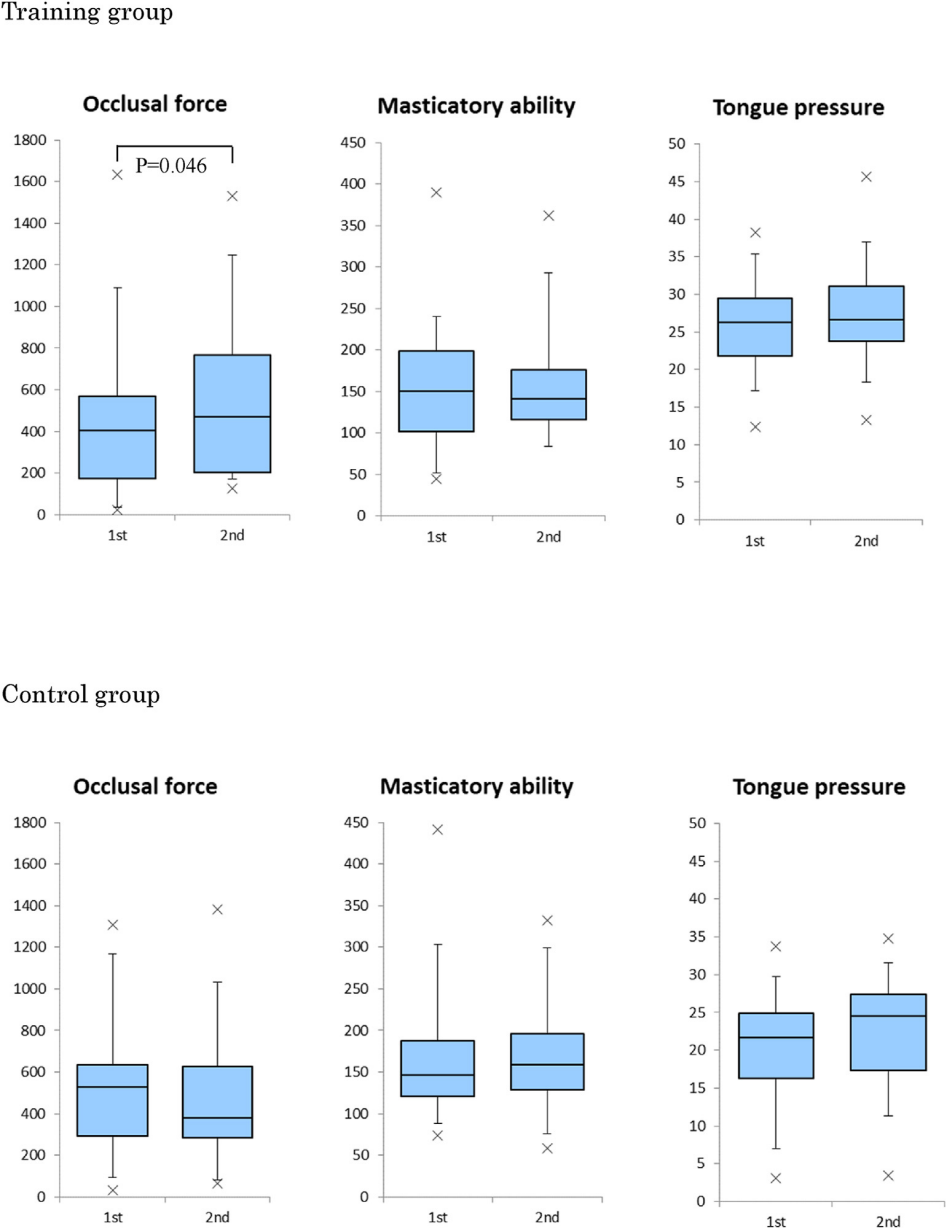
Changes in the occlusal force, masticatory ability, and tongue pressure after masticatory training. The occlusal force in the training group increased significantly from 404.2 (quartile: 173.5–567.7) N before training to 472.2 (200.8–766.3) N after training (*P* = 0.046). However, there were no significant differences in the masticatory ability (*P* = 0.896) or tongue pressure (*P* = 0.274). The control group showed no significant differences in any of the evaluation items (occlusal force: *P* = 0.178, masticatory ability: *P* = 0.952, and tongue pressure: *P* = 0.067) between the two measurement points.

Fig. 3 shows the changes in the occlusal force, masticatory ability, and tongue pressure between the two measurement points. The occlusal force in the training group increased significantly from 404.2 (quartile: 173.5–567.7) N before training to 472.2 (200.8–766.3) N after training (*P* = 0.046). However, there were no significant differences in the masticatory ability (*P* = 0.896) and tongue pressure (*P* = 0.274). Additionally, the control group showed no significant difference in all evaluation items (occlusal force: *P* = 0.178, masticatory ability: *P* = 0.952, and tongue pressure: *P* = 0.067) between the two measurement points.

## Discussion

In this study, the hardness of commercially available confectioneries was measured; masticatory training was conducted for 1 week using a confectionery in which the load was adjusted for each patient; and the effect of training on oral function was examined. As a result, the occlusal force of the patients in the training group increased significantly.

There are several reports on the effect of masticatory training using foods on oral function. Gummies and gums are found to be highly useful for analyzing the stability of masticatory movement pathways and rhythms; however, because their hardness and amount are constant, their use is inappropriate for analyzing masticatory function in the course from the start of mastication to swallowing and is also difficult for patients using dentures because of adhesiveness.^10^^,^^19^ In a study that evaluated masticatory function using rice crackers for masticatory training in patients with dysphagia who were ingesting a paste diet, it was reported that 92.4% of the patients were able to ingest them without aspiration.^20^ It is considered that this is because rice crackers or biscuits are easily mixed with saliva to form a bolus after being crushed by the teeth. Therefore, in this study, rice crackers or biscuits that are likely to form a bolus were adopted as a training diet.

Previous studies had not set an appropriate load amount of training foods for each participant but used training foods with certain properties for all participants.^5^, ^6^, ^7^, ^8^, ^9^, ^10^, ^11^, ^12^ In rehabilitation, it is important to apply a load higher than the normal activity intensity to strengthen the muscles and consequently to improve their function.^2^^,^^21^^,^^22^ The rating of perceived exertion (RPE) is recommended for setting the exercise intensity in the elderly population, even in general exercises.^21^, ^22^, ^23^ This method sets the strength that subjectively feels “slightly tough” as an appropriate load by regarding it as 50–70% of the maximum muscle strength. In this study, the hardness that the participants felt as “chewable but difficult to chew” was set to feel slightly tough based on the RPE; accordingly, the load value was suitable for boosting the masticatory muscle strength for the individuals. It has been reported that the masticatory muscle and occlusal force are highly related,^24^ and the load amount, training period, and frequency could be set appropriately because the occlusal force increased as a result of training in this study.

Several studies have shown that masticatory training improves the masticatory ability in the elderly population. One study in which masticatory training food (pure) was eaten three times, the level of mastication and swallowing increased.^7^ In another study in which novel chewable foodstuffs (gums) were eaten for 4 weeks, bite force and memory acquirement increased significantly.^11^ Another study showed that gum-chewing training for 30 days improved chewing ability and physical function.^12^ These previous studies had the elderly participants perform almost 4 weeks of training.^11^^,^^12^ In the present study, the training duration was limited to a week because many participants claimed fatigue and refused to eat a confectionery with a hardness adjusted individually as “chewable but difficult to chew” for more than a week. As the training foods were gum or gummy jerry in the previous studies, these foods required less effort to eat than those in this study, and it is considered the elderly participants could continue to train for 4 weeks. However, the present study suggested the occlusal force improved by the training even in a week.

The tongue pressure did not increase significantly in this study. This is because the load on the tongue might have been insufficient because rice crackers and biscuits are water-absorbent and tend to become boluses at an early stage when mixed with saliva. Further, the masticatory ability did not increase with masticatory training. In masticatory training, the increase in the maximum occlusal pressure and masticatory efficiency may not always be parallel. Another report stated that masticatory ability and body function were significantly improved 4 weeks after the start of masticatory training.^12^ It is possible that the training period is insufficient to improve the condition.

In this study, there were no differences in age, sex, Eichner classification, number of occlusal support areas with prosthetics mounted, and DSS score between the two groups. There were no factors besides masticatory training that affected the study results regarding patient masticatory and swallowing functions.

One limitation of this study is that the target of the load was 50–70% of the maximum muscle strength, while the actual setting of the load was determined by the subjective sensation of the patients. In the future, when an appropriate method for measuring the training load is established, it will be necessary to measure it accurately. Another limitation is that we focused only on the hardness of the confectioneries; however, the other physical properties of foods (adhesiveness and cohesiveness) differ among confectioneries, which can also affect masticatory function.

In conclusion, patients aged 65 years and older underwent masticatory training in which they ate a confectionery with a hardness adjusted individually for a week. A significant increase in the occlusal force was observed, suggesting that masticatory training using confectioneries with a hardness suitable for the individual is effective. In the future, it could be significant to also investigate whether the improved oral function is maintained after masticatory training is terminated.

## Declaration of competing interest

The authors declare no conflicts of interest with respective to the research and publication of this article.
