# Supplementary material for: Effect of masticatory training using confectioneries on oral function in elderly patients – A randomized controlled trial
Source: J Dent Sci. 2022 May 20;17(4):1480–6. doi: 10.1016/j.jds.2022.04.030 (PMC9588806; doi:10.1016/j.jds.2022.04.030)
Supplement: Supplementary file 2 [file mmc2.doc]

**
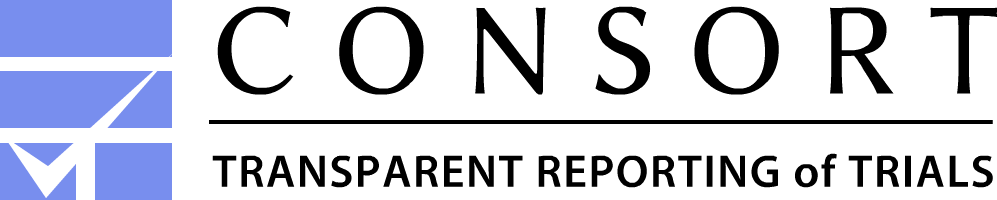
**

**CONSORT 2010 Flow Diagram**

**Allocation**

**Analysis**

**Follow-Up**

**Enrollment**

Assessed for eligibility (n=49)

Excluded (n=5)

♦  Not meeting inclusion criteria [dementia (n=3)]

♦  Declined to participate (n=2)

♦  Other reasons (n=0)

Analysed (n=21)
♦ Excluded from analysis (give reasons) (n=0)

Lost to follow-up [Lost of denture (n=1) and decline to participate (n=1)] Discontinued intervention (give reasons) (n=0)

Allocated to intervention (n=23)

♦ Received allocated intervention (received training with snacks) (n=23)

♦ Did not receive allocated intervention (give reasons) (n=0)

Lost to follow-up [Lost of denture (n=1) and worsen of disease (n=1)] Discontinued intervention (give reasons) (n=0)

Allocated to intervention (n=21)

♦ Received allocated intervention (not received training with snacks) (n=21)

♦ Did not receive allocated intervention (give reasons) (n=0)

Analysed (n=19)
♦ Excluded from analysis (give reasons) (n=0)

Randomized (n=44)
